# Questioning the role of HIPEC in patients with granulosa cell ovarian tumours

**DOI:** 10.1515/pp-2024-0002

**Published:** 2024-12-05

**Authors:** Christos Iavazzo, Ioannis D. Gkegkes

**Affiliations:** Gynaecological Oncology Department, Metaxa Cancer Hospital, Piraeus, Greece; Athens Colorectal Laboratory, Athens, Greece; Department of Colorectal Surgery, 9553 Royal Devon and Exeter NHS Foundation Trust, Exeter, UK

To the Editor,

With great deal of interest we read your article entitled “Peritoneal metastases from rare ovarian cancer treated with cytoreductive surgery and hyperthermic intraperitoneal chemotherapy (CRS/HIPEC)” by Falla-Zuniga et al. [1]. The authors present a single centre cohort of patients who had cytoreductive surgery and hyperthermic intraperitoneal chemotherapy for rare ovarian malignancies among which clear cell, endometrioid, granulosa cell, low-grade serous, mesonephric, mucinous, and small cell carcinomas. The authors highlight that less than 50 % of patients had disease recurrence after CRS/HIPEC, with higher rates of extra-vs. intra-peritoneal recurrences (50.0 vs. 25.0 %) and conclude that HIPEC can also provide loco-regional disease control in rare ovarian tumours [1].

We would like to focus on granulosa cell tumours and ask the authors for further clarifications regarding the HIPEC agents used and adjuvant chemotherapy recommendations. Three out of the 28 patients were patients with granulosa cell tumour with an age ranging from 51 to 76 years and a PCI score from 5 to 21. The used HIPEC agents were carboplatin, cisplatin and doxorubicin and melphalan. No information is provided regarding the adjuvant chemotherapy and the site of first recurrence. The PFS (progression free survival) was ranging from 0.5 to 31.7 months and similarly the OS (overall survival) from 0.5 to 31.7 months again. Could you please clarify why the PFS and OS is exactly the same? Moreover, there is no information whether such cases involved primary or recurrent disease.

Granulosa cell tumours are rare ovarian malignancies with a relatively favourable prognosis characterized by a tendency to late recurrences. Even in patients who develop recurrence, there is still no evidence to confirm the survival benefit of adjuvant chemotherapy if complete cytoreduction is achieved while it is a possible option for inoperable or suboptimally cytoreduced patients [2]. Data from Germany do not support the use of adjuvant chemotherapy in early and advanced stages of adult granulosa cell tumours [3]. A recent study showed that there is no benefit from the administration of adjuvant therapy following complete CRS for granulosa cell tumour relapse [4].

Once again, we would like to thank the authors for sharing their experience and we look forward to further information regarding the subgroup of patients with granulosa cell tumours.
